# Sequence-dependent trafficking and activity of GDE2, a GPI-specific phospholipase promoting neuronal differentiation

**DOI:** 10.1242/jcs.235044

**Published:** 2020-02-10

**Authors:** Fernando Salgado-Polo, Michiel van Veen, Bram van den Broek, Kees Jalink, Daniela Leyton-Puig, Anastassis Perrakis, Wouter H. Moolenaar, Elisa Matas-Rico

**Affiliations:** 1 Division of Biochemistry, Oncode Institute, The Netherlands Cancer Institute, Plesmanlaan 121, 1066 CX Amsterdam, The Netherlands; 2 Division of Cell Biology, The Netherlands Cancer Institute, Plesmanlaan 121, 1066 CX Amsterdam, The Netherlands; 3 Division of Biochemistry, The Netherlands Cancer Institute, Plesmanlaan 121, 1066 CX Amsterdam, The Netherlands

**Keywords:** Glycosylphosphatidylinositol, GPI-anchored protein, Glycerophosphodiester phosphodiesterase, Rab GTPase, Neurodegeneration, Neuroblastoma

## Abstract

GDE2 (also known as GDPD5) is a multispanning membrane phosphodiesterase with phospholipase D-like activity that cleaves select glycosylphosphatidylinositol (GPI)-anchored proteins and thereby promotes neuronal differentiation both *in vitro* and *in vivo*. GDE2 is a prognostic marker in neuroblastoma, while loss of GDE2 leads to progressive neurodegeneration in mice; however, its regulation remains unclear. Here, we report that, in immature neuronal cells, GDE2 undergoes constitutive endocytosis and travels back along both fast and slow recycling routes. GDE2 trafficking is directed by C-terminal tail sequences that determine the ability of GDE2 to cleave GPI-anchored glypican-6 (GPC6) and induce a neuronal differentiation program. Specifically, we define a GDE2 truncation mutant that shows aberrant recycling and is dysfunctional, whereas a consecutive deletion results in cell-surface retention and gain of GDE2 function, thus uncovering distinctive regulatory sequences*.* Moreover, we identify a C-terminal leucine residue in a unique motif that is essential for GDE2 internalization. These findings establish a mechanistic link between GDE2 neuronal function and sequence-dependent trafficking, a crucial process gone awry in neurodegenerative diseases.

This article has an associated First Person interview with the first author of the paper.

## Abstract (precis)

**Summary:** GDE2, a six-transmembrane GPI-specific phospholipase, undergoes constitutive endosomal recycling via newly identified sequences and a unique leucine residue in its C-terminal tail as a way to regulate neuronal differentiation.

## INTRODUCTION

Glycosylphosphatidylinositol (GPI)-anchored proteins at the plasma membrane are involved in the regulation of many vital biological functions, including signal transduction, cell adhesion, intercellular communication and differentiation. GPI anchoring is a complex post-translational modification that tethers membrane proteins, via their C-terminus, to a glycosylated phosphatidylinositol (PI) core in the outer leaflet of the plasma membrane, particularly at lipid raft nanodomains (Ferguson et al., 2015; Fujita and Kinoshita, 2010; Paulick and Bertozzi, 2008). Since they lack a transmembrane domain, GPI-anchored proteins cannot transmit signals by themselves but must interact with transmembrane effectors or cellular adhesion pathways to achieve signaling competence. Importantly, GPI anchoring confers a unique property to membrane proteins, namely susceptibility to phospholipase attack. Indeed, GPI-linked proteins can be released from their anchors and detected as soluble proteins in culture medium and body fluids. However, identification of the responsible phospholipase(s) and their biological function has long been elusive.

**Fig. 1.**
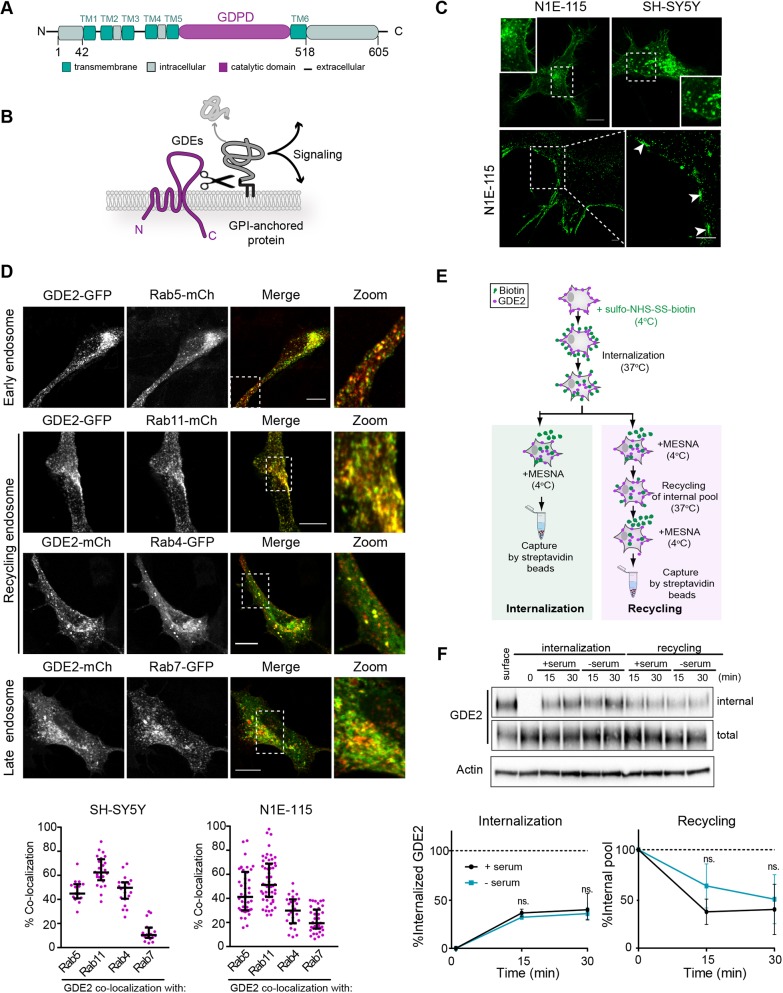
**GDE2 localization and endocytic trafficking routes.** (A) Domain structure of GDE2 showing six transmembrane (TM) domains, a GDPD ectodomain and intracellular N- and C-terminal tails. (B) GDE2 cleaves and sheds GPI-anchored proteins resulting in activation of signaling cascades. (C) GDE2 subcellular localization. Top, confocal images showing GDE2–GFP in membrane microdomains and intracellular vesicles in N1E-115 and SH-SY5Y cells. Bottom, super-resolution images of N1E-115 cells expressing GDE2–GFP. White arrows point to membrane microdomains. Scale bars: 10 μm (top), 1 μm (bottom). See also Matas-Rico et al. (2016). (D) Confocal images of GDE2 in early, recycling and late endosomes (Rab5-, Rab11-, Rab4- and Rab7-positive, respectively), in SH-SY5Y cells. Scale bars: 10 μm. Bottom panels show quantification of GDE2 colocalization with the indicated Rab GTPases, expressed as the percentage of yellow versus red pixels (≥25 cells from three independent experiments). Data represent the median±interquartile range of colocalization. (E) Schematic illustration of the internalization and recycling assay using biotin labeling. Cells expressing GDE2–mCh were surface-labeled with NHS-S-S-Biotin. Internalization proceeded for 15 and 30 min at 37°C in presence or absence of 10% FBS. Surface biotin was reduced with MesNa at 4°C, and the cells were shifted to 37°C for the indicated time periods to trigger recycling of the internal pool. (F) The amount of internalized and total biotin-labeled GDE2 in SH-SY5Y cells was determined by immunoblotting using anti-HA antibody. Actin was used as loading control. SH-SY5Y cells inducibly expressing GDE2–HA were surface-labeled with NHS-S-S-Biotin. Labeled GDE2 was allowed to internalize or recycle in the presence or absence of 10% FBS, as indicated. Representative western blots are shown as well as quantified band density from three independent experiments. Internalization was normalized to surface GDE2, and recycling to the internalized pool after 30 min. Data represent the mean±s.e.m. Results were not significantly different (ns) between with or without serum (one-way ANOVA). Similar results were obtained in N1E-115 cells (Fig. S2C).

Recent studies have identified members of the glycerophosphodiester phosphodiesterase (GDPD) family (Corda et al., 2014), notably GDE2 and GDE3 (also known as GDPD5 and GDPD2, respectively), as unique GPI-specific phospholipases that cleave select GPI-anchored proteins and thereby alter cell phenotype (Matas-Rico et al., 2016; Park et al., 2013; van Veen et al., 2017). GDE2 is by far the best studied family member and, along with GDE3 and GDE6 (GDPD4), is characterized by six transmembrane helices, a catalytic ectodomain and intracellular N- and C-terminal tails (Fig. 1A,B). GDE2 acts in a phospholipase D (PLD)-like manner, as inferred from its ability to release choline from glycerol-3-phosphocholine *in vitro* (Gallazzini et al., 2008). GDE2 was originally found to drive neuronal maturation and survival in the developing spinal cord (Rao and Sockanathan, 2005; Sabharwal et al., 2011) through cleavage of GPI-anchored RECK, a Notch ligand regulator, leading to Notch inactivation and induction of neurogenesis in adjacent neural progenitors (Park et al., 2013); in this way, GDE2 thus acts in a non-cell-autonomous manner. In marked contrast to GDE2, its close relative GDE3 functions as GPI-specific phospholipase C (PLC) and shows different substrate preference from GDE2, the structural basis of which remains unclear (van Veen et al., 2017). In the brain, GDE2 is mainly expressed in neurons and oligodendrocytes, whereas GDE3 expression is restricted to astrocytes, indicative of cell type-specific signaling functions (https://web.stanford.edu/group/barres_lab/brain_rnaseq.html).

We recently reported that GDE2 promotes neuronal differentiation in a cell-autonomous manner through *cis*-cleavage of glypican-6 (GPC6) (Matas-Rico et al., 2016). Glypicans (GPC1–GPC6) are GPI-anchored heparan sulfate proteoglycans (HSPGs) that play key roles in morphogenesis, and can influence signaling cascades and biological functions via various mechanisms, including growth factor recruitment or direct binding to transmembrane receptors such as type-II receptor protein phosphatases (RPTPs) (Capurro et al., 2017; Farhy-Tselnicker et al., 2017; Ko et al., 2015). Enforced GDE2 expression led to altered Rho GTPase signaling, upregulation of neuronal differentiation markers, neurite outgrowth and resistance to neurite retraction through an as-yet-unknown transmembrane effector pathway (Matas-Rico et al., 2016). Moreover, *GDE2* expression strongly correlated with positive clinical outcome in neuroblastoma (Matas-Rico et al., 2016), an often lethal neurodevelopmental malignancy characterized by impaired differentiation (Ratner et al., 2016). Importantly, *Gde2*-knockout in mice leads to progressive neurodegeneration with pathologies reflecting human neurodegenerative disease, which was accompanied by reduced glypican release, implicating dysregulated GPI-anchored protein activity in neurodegeneration (Cave et al., 2017). Finally, GDE2 depletion in zebrafish embryos resulted in motility defects and impaired pancreas differentiation, as shown by reduced insulin expression, which was attributed in part to altered Notch regulation (van Veen et al., 2018). Expression of human GDE2 restored insulin expression in these embryos, indicating functional conservation between zebrafish and human GDE2 (van Veen et al., 2018). Taken together, these results underscore the need for tight control of GDE2 surface expression and activity *in vivo*. However, it remains unknown how GDE2 is regulated.

Here, we report that, in undifferentiated neuronal cells, GDE2 is regulated by membrane trafficking under control of its long C-terminal tail. Membrane trafficking is of vital importance for cellular homeostasis and numerous signaling processes, particular in the nervous system, and its dysregulation can lead to disease. We identify distinctive C-terminal sequences that govern GDE2 exocytosis, endocytosis and recycling preference, and thereby modulate GDE2 biological activity both positively and negatively. In particular, through consecutive truncations, GDE2 recycling preference and cell-surface expression can be manipulated to render GDE2 either dysfunctional or hyperactive. Importantly, we discover a leucine residue in a non-canonical sorting motif that is key for GDE2 endocytosis and ensuing recycling. Together, our results reveal the sequence determinants of GDE2 trafficking and activity, thus providing mechanistic insight into cell-intrinsic GPI-anchor cleavage and its (neuro)biological outcome. Since membrane trafficking is impaired in neurodegenerative diseases, while GDE2 protects against neurodegeneration in mice, our findings may have pathophysiological implications, as will be discussed.

## RESULTS

### GDE2 trafficking – constitutive endocytosis and recycling

Initial pilot studies showed that in undifferentiated neuronal cells, GDE2 is enriched in recycling endosomes, strongly suggesting regulation by membrane trafficking (Matas-Rico et al., 2016). We analyzed GDE2 trafficking in SH-SY5Y and N1E-115 neuronal cells, which resemble immature sympathetic neurons and express very low levels of endogenous GDE2 (Matas-Rico et al., 2016). GDE2 [tagged with HA, GFP or mCherry (mCh)] was detected in discrete microdomains or clustered nanodomains, as revealed by super-resolution microscopy (Matas-Rico et al., 2016; Fig. 1C). Intracellularly, GDE2 was particularly abundant in perinuclear vesicles (Fig. 1C). Of note, untagged GDE2 showed similar subcellular localization to tagged versions of GDE2 in both neuronal cell lines (Fig. S1A). GDE2–GFP-containing vesicles in single SH-SY5Y cells were highly mobile and show rapid bi-directional movement towards and from the neurite ending, as shown by live-imaging (Movie 1). Treatment with the dynamin inhibitor dynasore resulted in GDE2 accumulation at the plasma membrane with almost complete loss of GDE2-positive vesicles (Fig. S1B). Furthermore, we confirmed that GDE2 colocalized with the endogenous transferrin receptor (TfR), a prototypic cargo of the clathrin-dependent endocytosis route (Fig. S1C). From these results, we conclude that GDE2 undergoes classical clathrin- and dynamin-mediated internalization (Mettlen et al., 2018). GDE2 internalization via a parallel clathrin-independent pathway cannot formally be excluded, but is unlikely to make a substantial contribution since more than 90% of the earliest detectable endocytic vesicles in mammalian cells arise from clathrin-coated pits (Bitsikas et al., 2014). Finally, it is worth noting that endosomal GDE2 lacks signaling activity as its catalytic ectodomain is exposed to the vesicle lumen, not to the cytoplasm.

Rab GTPases are highly specific master regulators of membrane trafficking and play key roles in maintaining neuronal function (Mignogna and D'Adamo, 2018; Wandinger-Ness and Zerial, 2014). In both neuronal cell lines, GDE2–GFP colocalized with the early endosome marker Rab5–mCh (Fig. 1D; Fig. S2A), but the majority of intracellular GDE2 was detected in the Rab4- and Rab11-regulated recycling routes, known as the ‘fast’ and ‘slow’ routes, respectively (Fig. 1D; Fig. S2A). Rab4 (Rab4a and Rab4b)-mediated fast recycling involves a half-time of a few minutes, whereas Rab11 (Rab11a and Rab11b) regulates slow recycling with a half-time of >10 min (Maxfield and McGraw, 2004). A small proportion of GDE2 was detected in Rab7-positive (Rab7^+^; Rab7a and Rab7b) late endosomes, which deliver cargo to lysosomes for degradation, and a small fraction of GDE2 was indeed found in LAMP1-positive lysosomes (Fig. S3). Quantification of the results from both neuronal cell lines showed that GDE2 predominantly localized to Rab11^+^ endosomes (mean ∼60%), somewhat less to Rab5^+^ and Rab4^+^ endosomes (mean 30–45%, depending on the cell line), and much less to Rab7^+^ late endosomes and lysosomes (Fig. 1D; Fig. S3). In support of the colocalization results, biochemical studies using co-transfected HEK293 cells showed GDE2 formed a complex with Rab4, Rab5, Rab7 and Rab11 (Fig. S2B).

We measured GDE2 internalization and recycling in both neuronal cell lines using a biotin labeling procedure, in which biotinylated GDE2–HA was allowed to internalize at 37°C in the presence or absence of serum. The biotin moiety was then removed from the cell-surface GDE2 pool using the membrane-impermeant reducing agent MESNa at 4°C. Internalized GDE2 was triggered to recycle back by a temperature shift from 4°C to 37°C (Fig. 1E). As shown in Fig. 1F and Fig. S2C, internalized GDE2 was found to travel back to the plasma membrane within 15 to 30 min in both SH-SY5Y and N1E-115 cells, independent of serum stimulation. We therefore conclude that GDE2 endocytosis and recycling is a constitutive process and insensitive to serum factors.

### C-terminal tail truncations uncover unique regulatory sequences

**Fig. 2.**
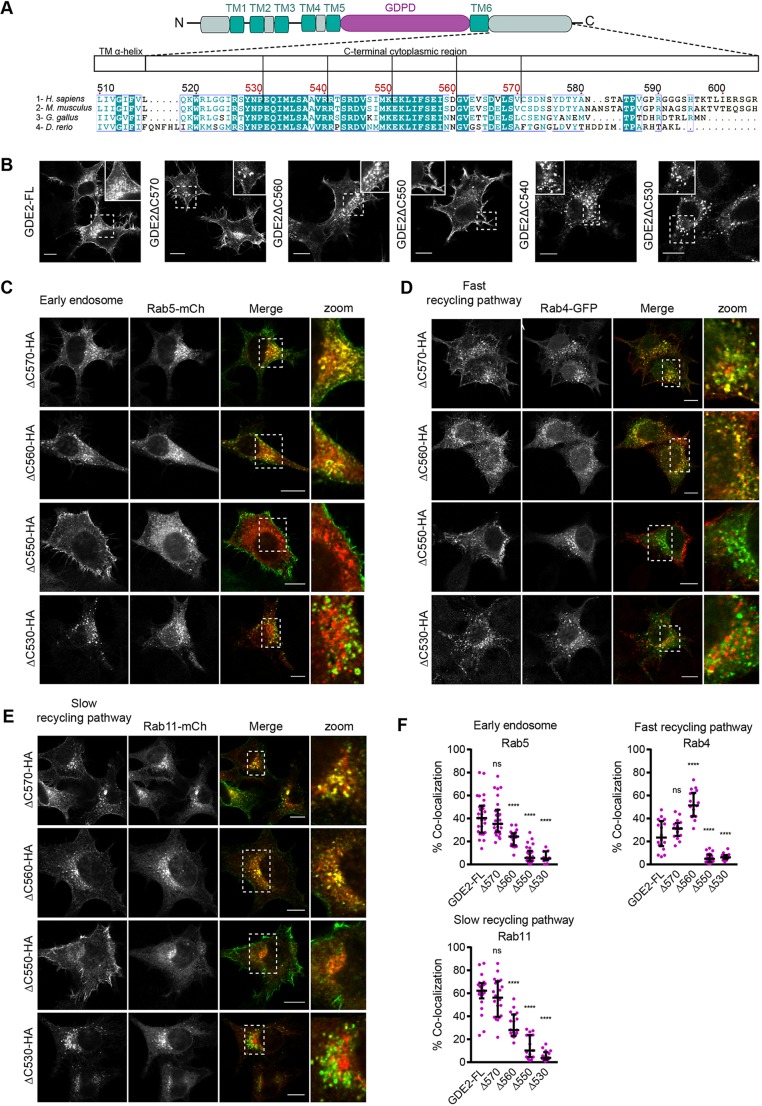
**C-terminal tail truncations uncover unique regulatory sequences.** (A) C-terminal sequence alignments of human, mouse, chicken and zebrafish GDE2, and a diagram of the truncation mutants in human GDE2 used in this paper. Note poor sequence conservation in the very C-terminal residues. (B) Subcellular localization of GDE2–HA and its truncation mutants in N1E-115 cells. (C–E) Confocal images of N1E-115 cells co-expressing the indicated GDE2 C-terminal truncations, and Rab5–mCh (C), Rab4–GFP (D) and Rab11–mCh (E). (F) Quantification of GDE2–Rab colocalization (percentage of yellow versus red pixels for ≥25 cells from three independent experiments). Data represent the median±interquartile range of colocalization. ****P<0.0001; ns, not significant (one-way ANOVA). Scale bars: 10 μm.

To explore the sequence determinants of GDE2 endocytosis and recycling, we focused on the long C-terminal tail [amino acids (aa) 518–605] (Fig. 2A). Motif searching did not reveal canonical tyrosine-based or acidic dileucine-based endocytic motifs (Bonifacino and Traub, 2003; Cullen and Steinberg, 2018), nor known protein interaction motifs. It is noteworthy that the distal C-terminal region (aa 570–605) of GDE2 shows marked sequence divergence among vertebrates (Fig. 2A), suggesting that these last 35 residues are not of major importance for GDE2 function but rather may play a species-specific role.

We made consecutive truncations in the C-terminal tail of GDE2 (Fig. 2A) and analyzed their colocalization with Rab5, Rab4 and Rab11 in N1E-115 cells. GDE2(ΔC570) showed the same subcellular localization as full-length GDE2, confirming that the last 35 residues are dispensable for proper GDE2 localization (Fig. 2B). GDE2(ΔC560) showed reduced surface expression (see also Fig. 4A,B below) and less colocalization with Rab11; remarkably, it accumulated preferentially in Rab4^+^ fast recycling endosomes at the expense of Rab11 colocalization (Fig. 2B–F). This suggests that the aa 561–570 region contains signaling information for balanced endocytic recycling of GDE2 (i.e. Rab4^+^ versus Rab11^+^ routing). GDE2(ΔC550), which has a further consecutive 10-aa truncation, is retained at the plasma membrane with no evidence of endosomal GDE2 (Fig. 2C–E), suggesting that the aa 551–560 stretch contains an essential endocytosis signal. Upon further truncation (at aa 540 or 530), GDE2 remained trapped intracellularly as aggregates (Fig. 2B–F), with GDE2(**Δ**C530) accumulating in lysosomes (Fig. S3). The respective GDE2–Rab protein colocalization results are quantified in Fig. 2F. We conclude that the aa 530–550 juxtamembrane region is required for proper GDE2 expression and exocytosis, while the aa 551–570 stretch contains all relevant trafficking information.

### Sequence determinants of GDE2 trafficking and cell surface expression

**Fig. 3.**
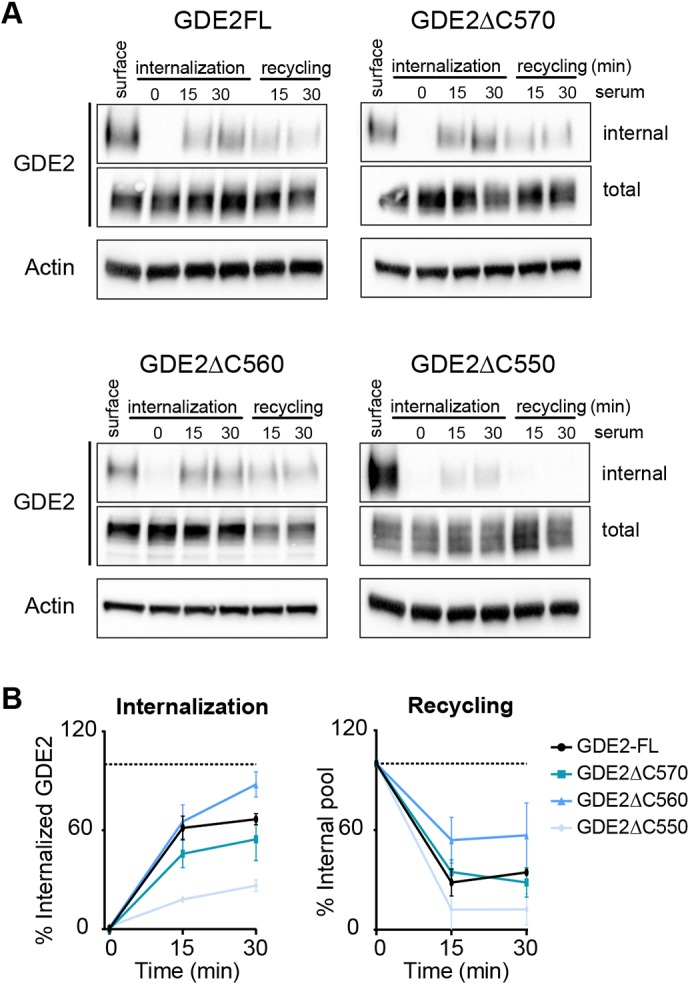
**C-terminal truncations impair internalization and recycling of GDE2.** (A) Internalization and recycling of GDE2-FL and its C-terminal truncations was followed during 30 min. SH-SY5Y cells inducibly expressing GDE2–HA (Fig. S4A) were surface-labeled with NHS-S-S-Biotin. Labeled GDE2 was allowed to internalize or recycle in the presence of 10% FBS as indicated. Representative western blots are shown. (B) Quantification of band density from three independent experiments. Internalization was normalized to surface GDE2, and recycling to the internalized pool after 30 min. Data represent the mean±s.e.m.

To determine the sequence determinants of GDE2 trafficking in further detail, we turned to SH-SY5Y cells stably expressing doxycycline (Dox)-inducible GDE2 (Matas-Rico et al., 2016). GDE2, its deletion mutants and catalytically dead GDE2(H233A) showed maximal expression after 24–48 h of Dox treatment; we also found that GDE2(**Δ**C540) and GDE2(**Δ**C530) were very poorly expressed (Fig. S4A). GDE2 trafficking was monitored during 30 min. in the presence of 10% serum using the biotin-labeling procedure (cf. Fig. 1E). As shown in Fig. 3A,B, GDE2-FL and GDE2(ΔC570) showed similar internalization and recycling kinetics, whereas recycling of the GDE2(ΔC560) internal pool was strongly reduced. By contrast, GDE2(ΔC550) underwent negligible internalization with no recycling. These results fully support those obtained in N1E-115 cells (Fig. 2).

**Fig. 4.**
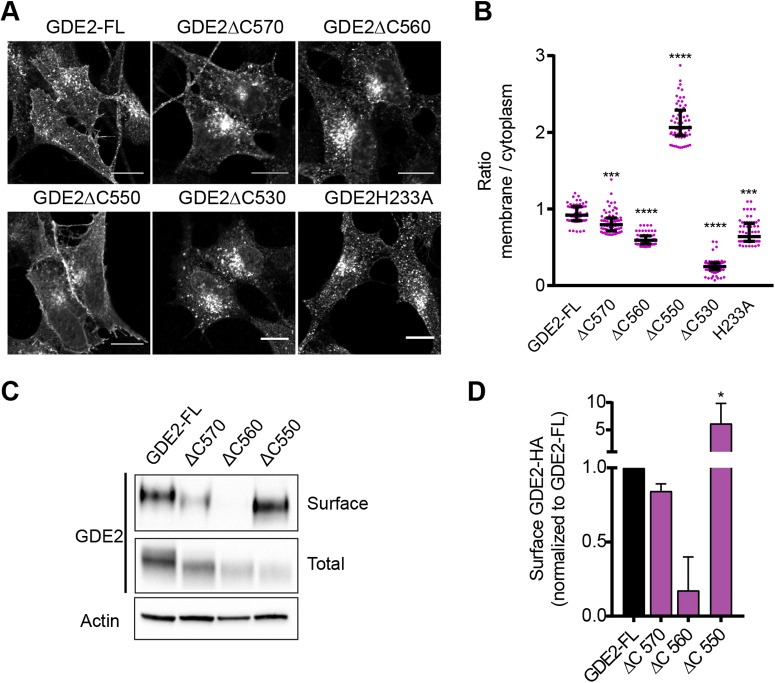
**Localization and trafficking of GDE2 and its C-terminal truncations.** (A) Localization of GDE2 and the indicated C-terminal truncation construct in Dox-treated SH-SY5Y cells (48 h). See also Fig. S4A. (B) Quantification of GDE2 surface versus cytosol localization in inducible SH-SY5Y cells (24 h). At least 20 cells from three independent preparations were segmented and analysed by ImageJ to calculate the membrane:cytoplasmic signal ratio (median±interquartile range); ***P<0.001, ****P<0.0001 (one-way ANOVA). (C) SH-SY5Y cells inducibly expressing GDE2–HA were surface-labeled with NHS-S-S-Biotin, immunoprecipitated with streptavidin beads and analyzed by western blotting. (D) Quantification of band density for surface expression normalized to GDE2-FL (median±s.e.m.); *P<0.05 (one-way ANOVA).

After Dox treatment, the GDE2 truncation mutants showed a differential distribution between plasma membrane and endocytic vesicles that was very similar to that in N1E-115 cells (Fig. 4A). Quantitative microscopic analysis (Fig. 4B) showed that full-length GDE2 (GDE2-FL) and GDE2(**Δ**C570) displayed similar surface-to-cytoplasm ratios, while GDE2(ΔC550) showed a much higher ratio, in agreement with its retention at the plasma membrane. GDE2(ΔC560) showed a reduced ratio, as it preferentially accumulates in fast recycling endosomes (Fig. 4B). GDE2(ΔC530) showed virtually no expression at the plasma membrane, while catalytically dead GDE2(H233A) showed a surface-to-cytoplasm ratio identical to wild-type, confirming that GDE2 trafficking and surface expression are independent of catalytic activity (Matas-Rico et al., 2016).

**Fig. 5.**
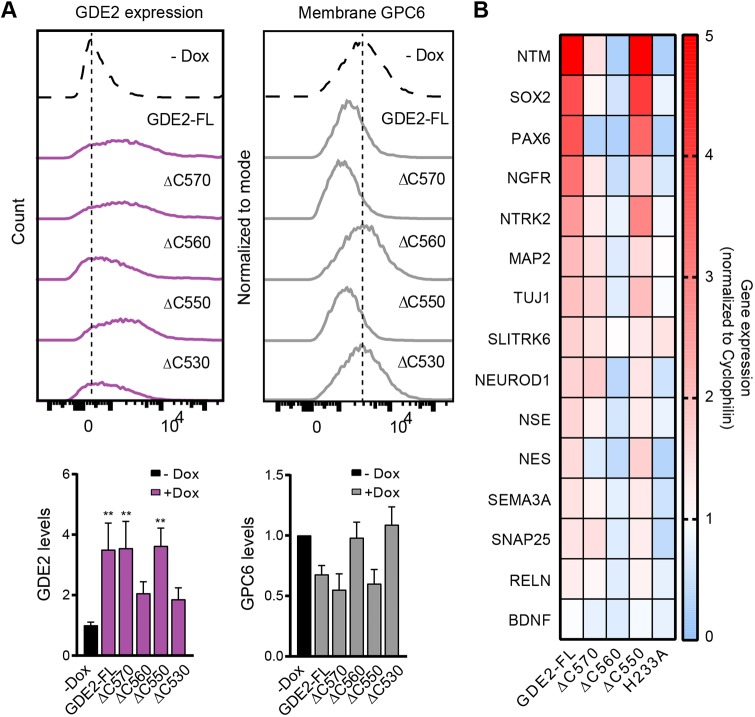
**Sequence-dependent trafficking determines GDE2 biological activity.** (A) Left panel, surface expression of GDE2–HA and its truncated mutants upon Dox-induced (48 h) expression, as detected by flow cytometry. Right panel, GPC6 surface levels as a function of GDE2–HA expression compared to that in GDE2-deficient cells (−Dox), as detected by flow cytometry using an anti-GPC6 antibody. In both cases, representative histograms from the same experiment are shown. Lower panels, quantifications (mean±s.e.m.) of the above FACS data from three independent experiments. **P<0.001 (one-way ANOVA). (B) Induction of neuronal differentiation marker genes upon Dox-induced expression of the indicated GDE2 constructs as determined by RT-qPCR and shown in a heat map. For quantification see Fig. S4C.

To quantify the relative surface expression levels, we biotin labeled cell surface GDE2–HA, which was then immunoprecipitated and detected by western blot. Analysis of the ratio of surface expression over total protein expression confirmed that GDE2 (ΔC570) behaved like GDE2-FL while GDE2(ΔC560) surface levels were strongly decreased when compared to GDE2-FL (Fig. 4C,D). Finally and consistent with the above results, cell surface expression of endocytosis-incompetent GDE2(ΔC550) was strongly enriched. We also measured the expression of GDE2 truncation mutants by flow cytometry (FACS) (Fig. 5A), using catalytically dead GDE2(H233A) as control (Fig. S4B). In agreement with the biotin-labeling results, GDE2-FL and GDE2(ΔC570) showed similar cell surface levels while GDE2(ΔC560) expression was reduced (Fig. 5A, left panel). However, this analysis did not reveal enhanced plasma membrane expression of GDE2(ΔC550), which we attribute to limitations inherent to FACS.

### Sequence-dependent trafficking determines GPC6-shedding activity of GDE2

To assess how GDE2 trafficking relates to activity, we used the above FACS conditions to measure GDE2 catalytic activity towards endogenous GPC6, a preferred GPI-anchored substrate of GDE2 in neuronal cells (Matas-Rico et al., 2016) (Fig. 5A; Fig. S4B). Catalytic activity of GDE2 and its truncation mutants towards GPC6 correlated with their respective surface levels. However, GDE2(**Δ**C560) was dysfunctional, as it failed to cause shedding GPC6 from SH-5Y5Y cells (Fig. 5A, left panel). Interestingly, this apparent loss of GPC6-shedding activity correlates with the preference of GDE2(**Δ**C560) for fast recycling in combination with its reduced expression at the plasma membrane. Our efforts to detect GDE2-induced GPC6 accumulation in the medium were unsuccessful because of high background levels of GPC6 in SH-SY5Y cells.

We therefore used a different method to measure GDE2-induced GPC6 accumulation release in the medium. For this, we used transiently transfected HeLa cells co-expressing mutant GDE2–HA and GPC6–GFP (Fig. S5). HeLa cells express low levels of endogenous GPC6 and show similar subcellular localizations of GDE2–HA and its mutants to that observed in neuronal cells (Fig. S5A). Cell lysates and supernatants were analyzed for GPC6–GFP by western blotting (Fig. S5B). The quantified results (Fig. S5C) indicate that endocytosis-incompetent GDE2(ΔC550) now showed a nearly two-fold higher GPC6-shedding efficacy than GDE2-FL, indicative of ‘hyperactivity’ and in agreement with its greater abundance on the plasma membrane. On the other hand, GDE2(ΔC560) was not completely dysfunctional towards GPC6 shedding under these alternate assay conditions.

### GDE2 regulation of neuronal differentiation genes

GDE2 regulates a neural differentiation transcriptional program, as shown by overexpression and knockdown studies (Matas-Rico et al., 2016). Building on those results, we investigated how GDE2 truncation affects the expression of select neuronal differentiation marker genes, including those encoding key transcription factors (SOX2, PAX6, NEUROD1), neurotrophin receptors (NGFR, NTRK2 and SLITRK6), presynaptic t-SNARE protein SNAP25, GPI-anchored neurotrimin (NTM), neuron-specific enolase (NSE) and cytoskeletal proteins (NES, MAP2 and TUJ1) and others. Induction of these genes by the respective GDE2 truncation mutants mirrored the induction of GPC6 release, albeit to varying degrees (Fig. 5B; Fig. S4C). GDE2(ΔC570) lacked full signaling activity, although it showed wild-type trafficking behavior and activity towards GPC6. Remarkably, however, GDE2(ΔC560) showed again loss-of-function as it failed to induce significant gene transcription similar to that seen with catalytically dead GDE2(H233A) (Fig. 5B; Fig. S4C). Finally, GDE2(ΔC550) showed virtually wild-type activity towards the differentiation markers, indicating full recovery of function that was lost in GDE2(ΔC560). The signaling pathways that lead from GDE2 surface activity to gene transcription remain to be explored.

### Residue Leu-553 dictates GDE2 endocytosis and cell surface expression

**Fig. 6.**
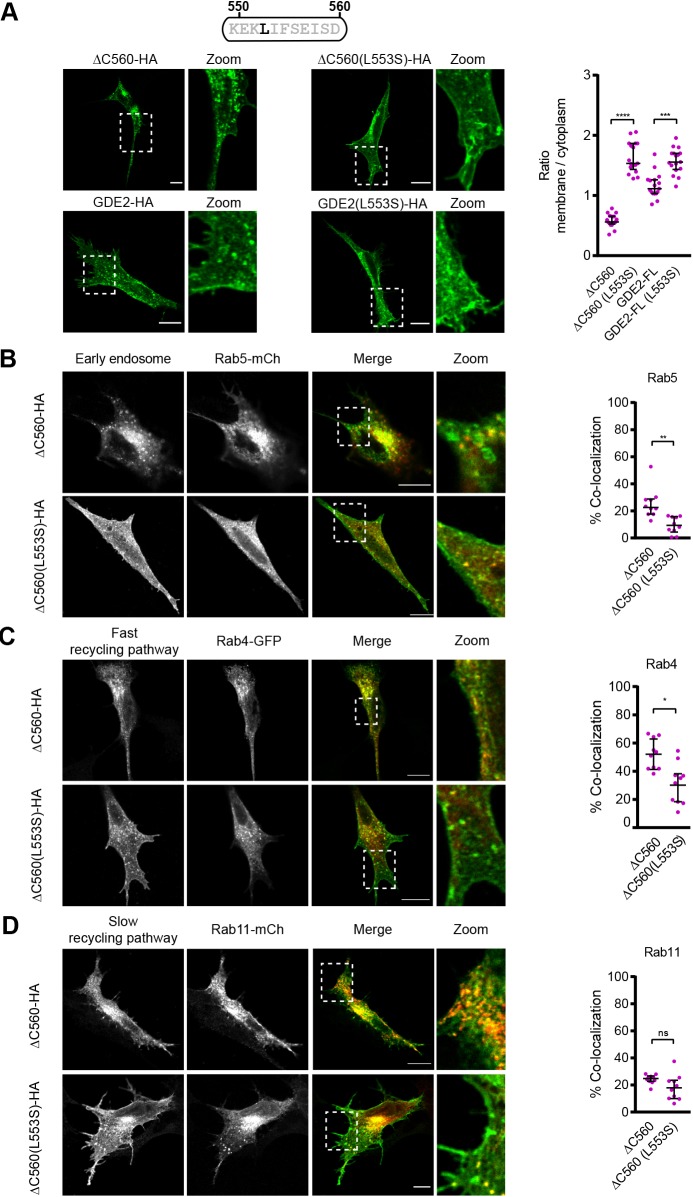
**Residue Leu-553 dictates endocytosis and cell-surface expression of GDE2(Δ560) and GDE2-FL.** (A) Left panel, mutating Leu-553 in GDE2(ΔC560) and GDE2-FL leads to increased plasma membrane localization in SH-SY5Y cells. Right panel, quantification of surface versus cytosol localization the mutated (L533S) and non-mutated GDE2 constructs in SH-SY5Y cells; 15 cells were segmented and analyzed with ImageJ to calculate the membrane:cytoplasmic signal ratio (median±interquartile range). ***P<0.001, ****P<0.0001 (one-way ANOVA). (B–D) Left panels, confocal images of SH-SY5Y cells co-expressing the indicated GDE2 mutants and Rab5–mCh (B), Rab4–GFP (C) and Rab11–mCh (D). Right panels, quantification of GDE2–Rab colocalization (percentage of yellow versus red pixels for 10 cells). Data represent the median±interquartile range of colocalization. *P<0.05, **P<0.01 (one-way ANOVA). Scale bars: 10 μm.

**Fig. 7.**
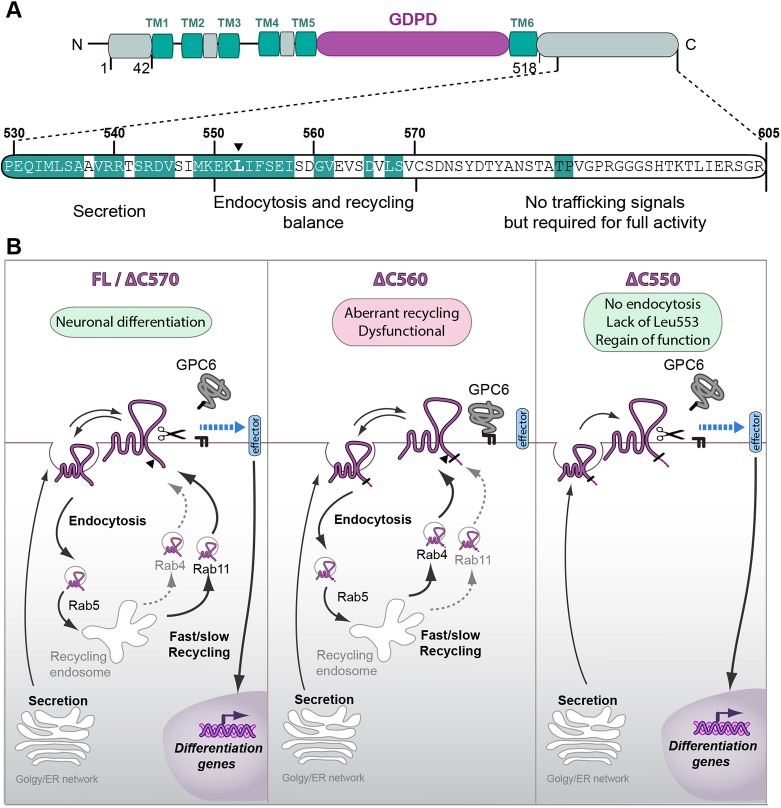
**Sequence determinants of GDE2 trafficking and biological activity.** (A) The C-terminal region (aa 530–550 or 541–550) is essential for proper expression, secretion and membrane insertion. The sequence from aa 551–560 determines endocytosis (with a key role for residue Leu-553, indicated by an arrowhead) and Rab4 fast recycling preference, but negatively regulates GDE2 function. Sequence from aa 561–570 is required for proper GDE2 recycling and function. Residues in white are not conserved between mammalian, chicken and/or zebrafish GDE2 (cf. Fig. 2A). (B) Scheme of membrane trafficking, localization and biological output of GDE2 and the indicated truncation mutants, acting in a cell-autonomous manner through GPC6 cleavage and an as-yet-unknown transmembrane effector. GDE2 is constitutively internalized while the majority of endocytosed GDE2 recycles along Rab4^+^ and Rab11^+^ routes in a sequence-dependent manner. A small percentage of internalized GDE2 is sorted to Rab7^+^ late endosomes and lysosomes (not illustrated). GDE2(ΔC570) shows wild-type trafficking behavior and GPC6-releasing activity, yet is not fully signaling competent. GDE2(ΔC560) shows preference for Rab4-driven fast recycling and is dysfunctional, whereas GDE2(ΔC550) is retained at the cell surface with regain of function. The endocytosis regulatory residue Leu-553 is indicated by a black arrowhead. Signaling efficacy is inferred from GPC6 shedding and induction of neuronal differentiation marker genes. See text for further details.

We next set out to determine what is unique about the aa 551–570 region that confers endocytosis and recycling on GDE2. Sequence inspection revealed a potential trafficking determinant, namely residue Leu-553, located in a putative non-canonical LI-based sorting motif (Fig. 6A). Canonical dileucine motifs are acidic and contain six amino acids (consensus [D/E]xxxL[L/I]), and mediate endocytosis of transmembrane proteins via direct interaction with the clathrin adaptor complex AP-2 (Bonifacino and Traub, 2003; Cullen and Steinberg, 2018; Guardia et al., 2018; Kelly et al., 2008). Mutating Leu-553 to a serine residue in GDE2(ΔC560) or GDE2-FL [which may have a more prominent effect than a leucine to alanine mutation in this case (Denzer et al., 1997)] led to strongly disrupted internalization and enhanced accumulation on the cell surface (Fig. 6A,B), and showed reduced colocalization with Rab5, Rab4 and Rab11 (Fig. 6B–D). Hence, Leu-553 is a key endocytosis regulatory residue that directs GDE2 into early endosomes (Rab5^+^) and ensuing recycling routes. Interestingly, the putative 6-aa LI-based endocytic motif (MKEKLI) is basic rather than acidic (Fig. 7A) and highly conserved among vertebrates (Fig. 2A). Mutating residues adjacent to Leu-553 should disclose the nature of this intriguing putative LI-based sorting motif.

## DISCUSSION

In this study, we have uncovered C-terminal tail sequences that regulate the trafficking and activity of GDE2, a unique GPI-specific phospholipase that promotes neuronal differentiation and, furthermore, is implicated in pancreas development. Moreover, we have identified a C-terminal leucine residue that is essential for GDE2 internalization. In neuronal cells, GDE2 undergoes constitutive endocytosis and recycling, independently of its catalytic activity or the presence of serum factors. GDE2 is internalized in a Dynasore-sensitive manner and co-traffics with the transferrin receptor. Hence, we conclude that GDE2 follows the classical clathrin-mediated endocytosis route (Mettlen et al., 2018), supported by the fact that clathrin-independent endocytosis plays only a minor role in mammalian cells (Bitsikas et al., 2014). GDE2 is then routed to Rab5^+^ early endosomes where sorting decisions are made. The large majority of GDE2 is recycled back to the cell surface along two distinct pathways, the fast (Rab4^+^) and slow (Rab11^+^) route, respectively. In general, constitutive recycling serves to generate a highly dynamic internal pool of membrane receptors or ecto-enzymes that is critical for their cell surface expression, signaling fidelity and ensuing cellular responses (Cullen and Steinberg, 2018). In case of GDE2, its constant recycling may serve to probe the cell surface for specific GPI-anchored substrates, such as GPC6, in a highly dynamic manner.

The C-terminal determinants of GDE2 trafficking, surface expression and activity were inferred from Rab GTPase colocalizations, GPC6 shedding and induction neural differentiation markers, respectively. Our major findings are summarized in Fig. 7. In brief, the last 35 C-terminal residues – which are poorly conserved among vertebrates (Fig. 2A) – are dispensable for GDE2 trafficking and enzymatic activity towards GPC6, yet the GDE2(ΔC570) mutant is not fully signaling competent in terms of biological activity. This suggests that the very C-terminal region contains trafficking-unrelated signaling information that is possibly species specific. Strikingly, upon two further 10-aa truncations, the resulting GDE2(ΔC560) mutant is redirected from slow (Rab11^+^) to fast (Rab4^+^) recycling with virtual loss of function, whereas GDE2(ΔC550) shows endocytosis failure, cell surface retention and (re)gain of function, as illustrated in the schemes of Fig. 7B. Finally, the juxta-membrane region at aa 518–550 appears to be required for GDE2 biosynthesis and export, as the ΔC540 and ΔC530 mutants are detected as aggregates and fail to enter the secretory pathway.

The most relevant regulatory sequence emerging from our analysis is the 20-amino-acid region at aa 551–570, as it contains all the signaling information essential for GDE2 endocytosis, proper recycling and activity. Of special importance is our identification of Leu-553 as key residue essential for GDE2 endocytosis and ensuing recycling. It is located in a highly conserved LI-based sequence (549-MKEK**LI**FSEI-558) (Fig. 7A) that does not conform to the consensus dileucine sorting motif, [D/E]xxxL[L/I] (Bonifacino and Traub, 2003; Cullen and Steinberg, 2018; Sanger et al., 2019). Mutating Leu-553 to a serine residue led to marked disruption of GDE2 internalization, with strongly reduced Rab GTPase colocalizations, and accumulation on the plasma membrane. In other words, mutating Leu-553 shifts the GDE2(ΔC560) phenotype to that of GDE2(ΔC550) (Fig. 7B). Dileucine-based motifs in the cytosolic tails of transmembrane proteins bind to the prototypical AP-2 clathrin adaptor complex that associates with the cytosolic face of the plasma membrane to mediate endocytosis (Kelly et al., 2008), with a strong requirement for inositol phospholipids (Sanger et al., 2019). Adaptor protein (AP) complexes such as AP-2 are particularly important for polarized sorting in neuronal cells, given their extreme morphological asymmetry (Bonifacino, 2014), while AP dysfunction underlies a range of neurological pathologies (Guardia et al., 2018; Sanger et al., 2019). Further studies are needed to define the exact sequence of this putative LI-based sorting motif and its possible interaction with the AP-2 complex.

We need to understand why GDE2(ΔC560) is dysfunctional in terms of defective GPC6 shedding and impaired induction of neuronal differentation markers. Owing to its preferred fast recycling, in combination with reduced cell surface expression, GDE2(ΔC560) may be misdirected to membrane nanodomains that are short of GPI-anchored substrates, particularly GPC6. Alternatively, accelerated recycling of GDE2(ΔC560) could entail a relatively short residence time at the plasma membrane with loss of effective GDE2–substrate interaction. For the ease of mechanistic reasoning, aberrant recycling and dysfunction of GDE2(ΔC560) is restored by adding another 10 residues to generate GDE2(ΔC570). In other words, GDE2(ΔC560) undergoes endocytosis because of Leu-533 but it lacks signals for balanced recycling and proper surface activity, which makes the adjacent 561–570 region particularly important in terms of regulation, even though this stretch is not well conserved among vertebrates (Figs 2A and 7A).

To gain further mechanistic insight it will be essential to identify adaptors and other binding partners of the GDE2 tail that not only drive the recycling machinery but may also regulate GDE2 activity independently of its trafficking behavior. In this respect, subcellular localization of GDE2 mediated by its transmembrane domains warrants further study (Cosson et al., 2013), as does its possible interaction with regulatory transmembrane proteins, such as, for example, the tetraspanins that associate with the ADAM10 ecto-protease to regulate its trafficking and substrate specificity (Matthews et al., 2017). Post-translational modifications might also modulate GDE2 trafficking and function, as exemplified by the finding that under oxidative conditions, disulfide bonding disrupts GDE2 exocytosis and cell-surface expression (Yan et al., 2015). However, we did not find evidence for GDE2 ubiquitylation or phosphorylation playing an important role in determining GDE2 subcellular localization [F.S.P., E.M.-R. and Roy Baas (Division of Biochemistry. The Netherlands Cancer Institute, Amsterdam), unpublished results].

Aside from tightly regulated trafficking, the biological outcome of GDE2 activity will critically depend on the local availability of GPI-anchored protein substrates and their spatiotemporal regulation in a given cell type. Determination of the substrate specificity for GDE2 and its structural basis therefore has a high priority. From a signaling point of view, another key question concerns the intracellular signaling routes by which GDE2-induced GPI-anchor cleavage triggers gene expression and neuronal differentiation cell autonomously. One attractive scenario implicates type-II receptor protein tyrosine phosphates (RPTPs) as candidate effectors, some of which exhibit high-affinity interactions with glypicans and might initiate new signaling events upon GDE2-incuded glypican cleavage (Coles et al., 2015; Ko et al., 2015).

### Potential pathophysiological implications

On a final note, GDE2 deficiency leads to progressive neurodegeneration in mice with motor neuron pathologies analogous to those in human disease, while it strongly correlates with poor clinical outcome in neuroblastoma (Cave et al., 2017; Matas-Rico et al., 2016). This raises the intriguing possibility that GDE2 dysfunction may underlie aspects of neurodegenerative disease and/or contribute to the pathophysiology of neuroblastoma. However, to our knowledge, disease-associated GDE2 deficits have not been documented to date, neither in neurodegenerative disease nor in malignant neuroblastoma. Given the present findings, however, GDE2 dysfunction could well result from defective endocytic trafficking rather than from loss-of-function mutations. Indeed, impaired membrane trafficking is a hallmark of neurodegenerative diseases, including sporadic amyotrophic lateral sclerosis (ALS), Parkinson's and Alzheimer's disease, and involves Rab GTPase dysfunction, endosomal misrouting and disturbed intracellular transport (Agola et al., 2011; De Vos and Hafezparast, 2017; Kiral et al., 2018; McMillan et al., 2017; Parakh et al., 2018; Schreij et al., 2016; Tan and Gleeson, 2019; Xu et al., 2018). Although cause–effect relationships are not always evident, disease-associated trafficking defects, even if subtle, could compromise the neuroprotective and differentiation-promoting function of GDE2 and thereby contribute to disease. Unraveling the trafficking mechanisms of GDE2 – and those of its GPI-anchored substrates – should help us to better understand the regulation of GDE2 activity and its (patho)physiological implications in the nervous system and beyond.

## MATERIALS AND METHODS

### Cells

SH-SY5Y, N1E-115 and HEK293 cells (obtained from the ATCC) were grown in Dulbecco's modified Eagle's medium (DMEM) supplemented with 10% fetal bovine serum (FBS) at 37°C under 5% CO_2_. Antibodies used were against HA (3F10 from Roche Diagnostics; 1:1000); β-actin (AC-15 from Sigma; 1:10,000); mCherry (16D7 from Thermo Fisher Scientific; 1:1000); LAMP-1 (ab2900 from Abcam; 1 µg/ml) and GPC6 (LS-C36518 from LifeSpan Bioscience; 1:100). Allophycocyanin (APC)-conjugated anti-HA epitope tag antibody (cat. no. 901523, Biolegend, 1:200); EZ-Link™ Sulfo-NHS-biotin and Streptavidin–agarose resins were from Pierce, and GFP or mCherry Trap^®^ beads were from ChomoTek; Fugene 6 from Invitrogen.

### Plasmids and transfections

Human GDE2 cDNA was subcloned in pcDNA3.1 as described previously (Matas-Rico et al., 2016). Truncated versions of GDE2 were generated by amplification of full-length GDE2–HA or GDE2–mCherry using reverse primers for the last residues of each truncation. This was followed by a digestion with *BamH*I and *EcoR*V, after which the amplified inserts were cloned into digested and gel-purified pcDNA3.1, and selected with ampicillin. GDE2 point mutants were generated by site-directed mutagenesis using two complementary oligonucleotides with the desired mutated bases at the center of their sequences. A temperature gradient from 55 to 60°C was used during the PCR amplifications. The PCR products were digested with *Dpn*I and transformed into DH5-α competent bacteria and screened for the expected mutated bases.

### Confocal and super-resolution microscopy

Cells cultured on 24 mm, #1.5 coverslips were washed and fixed with 4% paraformaldehyde (PFA), permeabilized with 0.1% Triton X-100 and blocked with 5% BSA for 1 h. Incubation with primary antibodies was done for 1 h, followed by incubation with Alexa-conjugated antibodies for 45 min at room temperature. For confocal microscopy, cells were washed with PBS, mounted with Immnuno-Mount^TM^ (Thermo Fisher Scientific) and visualized on a LEICA TCS-SP5 confocal microscope (63× objective). Super-resolution imaging was done using an SR-GSD Leica microscope equipped with an oxygen scavenging system, as previously described (Matas-Rico et al., 2016). In short, 15,000 frames were taken in TIRF or EPI mode at 10 ms exposure time. Movies were analyzed and corrected using the ImageJ plugin Thunderstorm (http://imagej.nih.gov/ij/), followed by correction with an ImageJ macro using the plugin Image Stabilizer.

### Live imaging

Live-cell imaging of GDE2–GFP in SH-SY5Y cells was undertaken on a Leica TCS SP5 confocal microscope equipped with 63× oil immersion lens (NA 1.4; Leica, Mannheim, Germany). Coverslips were mounted on a metal ring system and exposed to buffer solution (140 mM NaCl, 5 mM KCl, 1 mM MgCl_2_, 1 mM CaCl_2_, 10 mM HEPES pH 7.2 and 10 mM glucose). Excitation was at 488 nm and emission was collected between 510 and 580 nm. Images were collected every 7.8 s for 40 min. For display as a movie, the image was cropped and displayed with the Fire LUT using open source Fiji software, version 1.52p.

### GDE2 plasma membrane localization

We performed image analysis for plasma membrane localization of HA-tagged GDE2 constructs by using Fiji/ImageJ. In brief, confocal images stained for GDE2–HA were segmented and analyzed using Fiji software and a macro that automated the process. First, images were thresholded with the MaxEntropy algorithm to delimit single cells and filtered by Gaussian Blur (radius=2) and smoothed for segmentation with a median radius of two. Using the Region of Interest manager on Fiji, the background was delimited by using the Li algorithm for thresholding. The cytoplasmic regions were selected by subtracting the plasma membrane thickness (fixed to 0.5 μm, but adjustable from a 0.2–5.0 range) and eroded with a pixel width of one to avoid having empty membranes in segmented cells. Next, the plasma membrane region was obtained by subtracting the background to the cytoplasmic region. Finally, the ratio of the membrane to cytoplasmic signals was calculated from the median value of these regions.

### Western blotting

For western blotting, cells were washed with cold PBS, lysed in NP-40 buffer (50 mM Tris-HCl pH 7.4, 150 mM NaCl, 2 mM EDTA, 1% NP-40, 0.25% sodium deoxycholate) supplemented with protease inhibitors and centrifuged (20,000 ***g*** for 15 min). Protein concentration was measured using a BCA protein assay kit (Pierce) and LDS sample buffer (NuPAGE, Invitrogen) was added to the lysate or directly to the medium. Equal amounts were loaded on SDS-PAGE pre-cast gradient gels (4–12% Nu-Page Bis-Tris, Invitrogen), followed by transfer to nitrocellulose membrane. Non-specific protein binding was blocked by 5% skimmed milk in TBST followed by incubation with primary antibodies were overnight at 4°C in TBST with 2.5% skimmed milk, and then secondary antibodies conjugated to horseradish peroxidase (DAKO, Glostrup, Denmark) for 1 h at room temperature. Proteins were detected using ECL western blot reagent.

### Biotin labeling

For quantification of GDE2 internalization and recycling, we used a biotin-labeling assay. GDE2–mCh-expressing N1E-115 cells were serum starved for 1 h, transferred to ice, washed in ice-cold PBS, and surface labeled at 4°C with 0.2 mg/ml NHS-SS-biotin (Pierce). For GDE2 internalization, cells were exposed to serum-free medium at 37°C for the indicated time periods. Cells were transferred to ice and washed with PBS, the remaining surface biotin was reduced with sodium 2-mercaptoethane sulfonate (MesNa), and the reaction was quenched with iodoacetamide (IAA) prior to cell lysis. For recycling assays, cells were labeled with biotin as above, and incubated in serum-free medium at 37°C for 30 min to allow internalization of GDE2. Cells were returned to ice, washed with PBS, and biotin was reduced using MesNa*.* Recycling of the internal GDE2 pool was induced by a temperature shift to 37°C for 0–30 min. Cells were returned to ice, washed with PBS and surface biotin was reduced by MesNa. MesNa was quenched by IAA and the cells were lysed. Biotin-labeled GDE2 was detected using Streptavidin beads and anti-mCh antibody.

### Immunoprecipitation

For co-immunoprecipitation of GDE2 and Rabs, HEK293T cells were plated on plastic dishes of 10 cm diameter and transient co-transfected with GDE2–mCh or GDE2–GFP, and Rab4a–GFP, Rab5a–mCh, Rab7a–GFP or Rab11a–mCh. Rab constructs were provided by Coert Margadant (Dept. Molecular and Cellular Hemostasis, Sanquin, the Netherlands) and Jacques Neefjes (Dept. Chemical Immunology, University of Leiden, the Netherlands). After 24 h cells were lysed using RIPA buffer. Protein concentration was determined using Protein BCA protein assay kit (Pierce). Immunoprecipitation was carried out incubating 500 μg–1 mg cytoplasmic extracts with GFP or mCherry Trap^®^ beads (ChromoTek) at 4°C for 1 h. Beads were washed three times and eluted by boiling in SDS sample buffer for 10 min at 95°C. Supernatants were applied onto an SDS gel and subjected to immunoblot analysis.

### Inducible GDE2 expression

SH-SY5Y cells with inducible expression of GDE2 constructs were generated using the Retro-X™ Tet-On^®^ Advanced Inducible Expression System (Clontech), as described previously (Matas-Rico et al., 2016). After retroviral transduction, the cells were placed under selection with G418 (800 mg/ml) supplemented with puromycin (1 μg/ml) for 10 days. GDE2 induction (in the presence of 1 μg/ml doxycycline) was verified by western blotting and confocal microscopy. Transient transfection was performed with Fugene 6 reagent (Invitrogen) according to the manufacturer's instructions.

### Flow cytometry

For GPC6 and GDE2–HA surface expression analysis, cells were grown in complete medium with 10% FCS with or without doxycycline. Cells were trypsinized into single-cell suspensions and then ∼8×10^5^ cells were incubated with 5 μl of anti-GPC6 antibody LS-C36518 (LifeSpan Bioscience) and in 4 μl of APC-conjugated anti-HA antibody (BioLegend). Bound GPC6 antibody were detected by incubating with a 1:200 dilution of goat Alexa-Fluor-488-conjugated anti-mouse-IgG secondary antibody in 2% BSA for 45 min on ice. Fluorescence measurements were performed using BD LSRFORTESSA and using FlowJo software.

### GDE2 enzymatic activity assays

GDE2 activity assays were carried out in HEK293 cells, essentially as described by Matas-Rico et al. (2016). HeLa cells were seeded on six-wells plates and co-transfected with expression vectors for human HA-tagged GDE2 or its truncations together with GFP-tagged GPC6. At 24 h after transfection with FuGENE^®^ 6, cells were incubated for an additional 24 h in DMEM with 0.1% FCS. The conditioned medium was removed and cell lysates were prepared using NP-40/NaDOC lysis buffer (50 mM Tris-HCl pH 7.4, 150 mM NaCl, 2 mM EDTA, 1% NP-40, 0.25% NaDOC and 5% glycerol) supplemented with protease inhibitor cocktail. The amount of substrate proteins in the medium and cell lysates was analyzed by western blotting.

### Induction of neural differentiation marker genes

Total RNA was extracted using the GeneJET purification kit (Fermentas). cDNA was synthesized by reverse transcription from 5 μg RNA using the First Strand cDNA Syntesis Kit (Thermo Fisher Scientific). Real-time quantitative PCR (RT-qPCR) was performed on a 7500 Fast System (Applied Biosystems) as follows: 95°C for 2 min, 95°C for 0 min, 60 cycles at 95°C for 15 s followed by 60°C for 1 min for annealing and extension. The final reaction mixtures (12 μl) consisted of diluted cDNA, 16SYBR Green Supermix (Applied Biosystems), 200 nM forward primer and 200 nM reverse primer. Reactions were performed in 384-well plates with three independent biological replicas. The primers used are listed in Table S1. As a negative control, the cDNA was replaced by milliQ water. Cyclophilin was used as reference gene. Each sample was analyzed in triplicate and the normalized expression (NE) data were calculated with the equation NE=2(Ct target−Ct reference).

## Supplementary Material

Supplementary information
