# Supplementary material for: Sequence-dependent trafficking and activity of GDE2, a GPI-specific phospholipase promoting neuronal differentiation
Source: J Cell Sci. 2020 Feb 10;133(3):jcs235044. doi: 10.1242/jcs.235044 (PMC7033719; doi:10.1242/jcs.235044)
Supplement: Supplementary information [file joces-133-235044-s1.pdf]

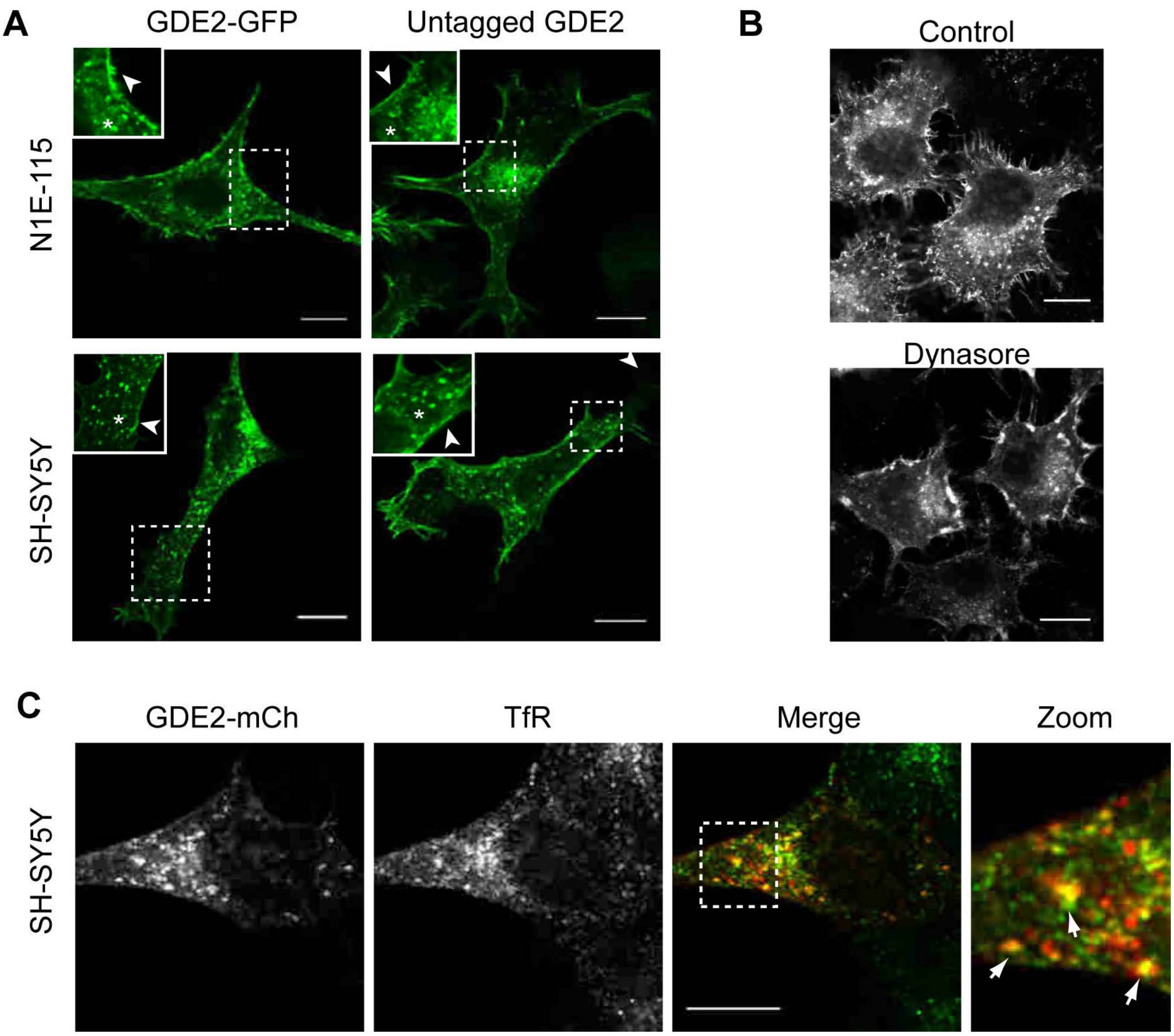

Figure S1. GDE2 subcellular localization and endocytic routes.

A. Confocal images showing GDE2-GFP and untagged GDE2 localization in membrane microdomains and intracellular vesicles in N1E-115 and SH-SY5Y cells. White arrows point to microdomains at the plasma membrane and asterisk to intracellular vesicles. Bar, 10 μm.

B. GDE2 accumulates at the cell surface with loss of GDE2-positive vesicles in N1E-115 cells treated with the dynamin inhibitor Dynasore (80 μM). Bar, 10 μm.

C. GDE2 co-localizes with endogenous transferrin receptor. SH-SY5Y cells were transiently transfected with GDE2-mCherry, fixed, and endogenous transferrin receptor (TfR) detected with an anti-TfR antibody. The zoom panel shows a magnified view of the area outline by the dashed square. White arrows point to GDE2- and TfR-positive vesicles. Bar 10 μm.

Journal of Cell Science • Supplementary information

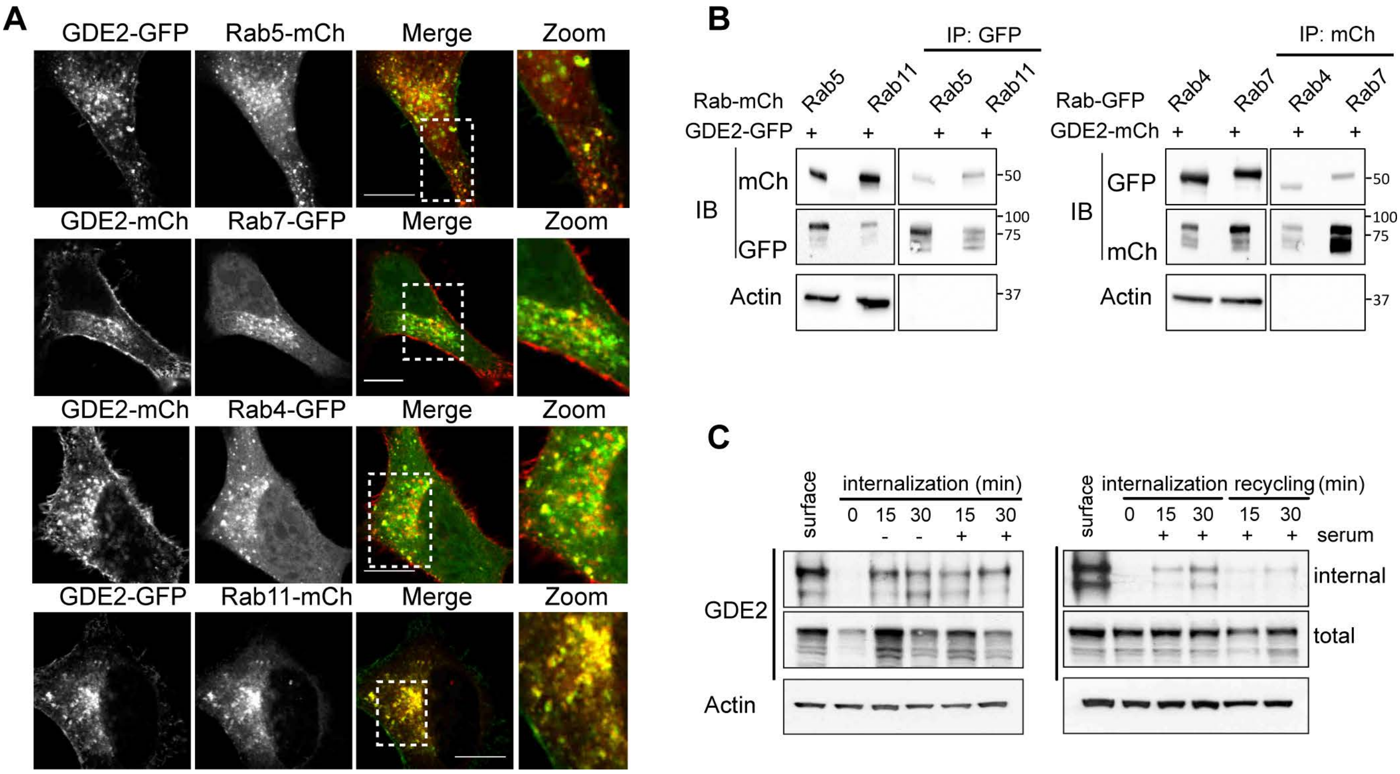

Figure S2. GDE2 colocalizes with Rab GTPases.

A. GDE2 co-localization with the indicated Rab GTPases in N1E-115 cells. Bar, 10 µm.

B. GDE2 (fused to GFP or mCh) associates with the indicated Rab GTPases in HEK293T cells. GDE2 was immunoprecipitated (IP) and subjected to immunoblotting (IB) using either anti-GFP or anti-mCherry antibody.

C. N1E-115 cells expressing GDE2-mCh were surface-labelled with NHS-S-S-Biotin. Internalization proceeded for 15 and 30 min at 37˚C in presenceor absence of 10% FBS. Surface biotin was reduced with MesNa at 4˚C, and the cells were shifted to 37˚C for the indicated time periods to trigger recycling of the internal pool. The amount of internalized and total biotin-labelled GDE2 determined by immuno-blotting using anti-mCh antibody. Actin was used as loading control.

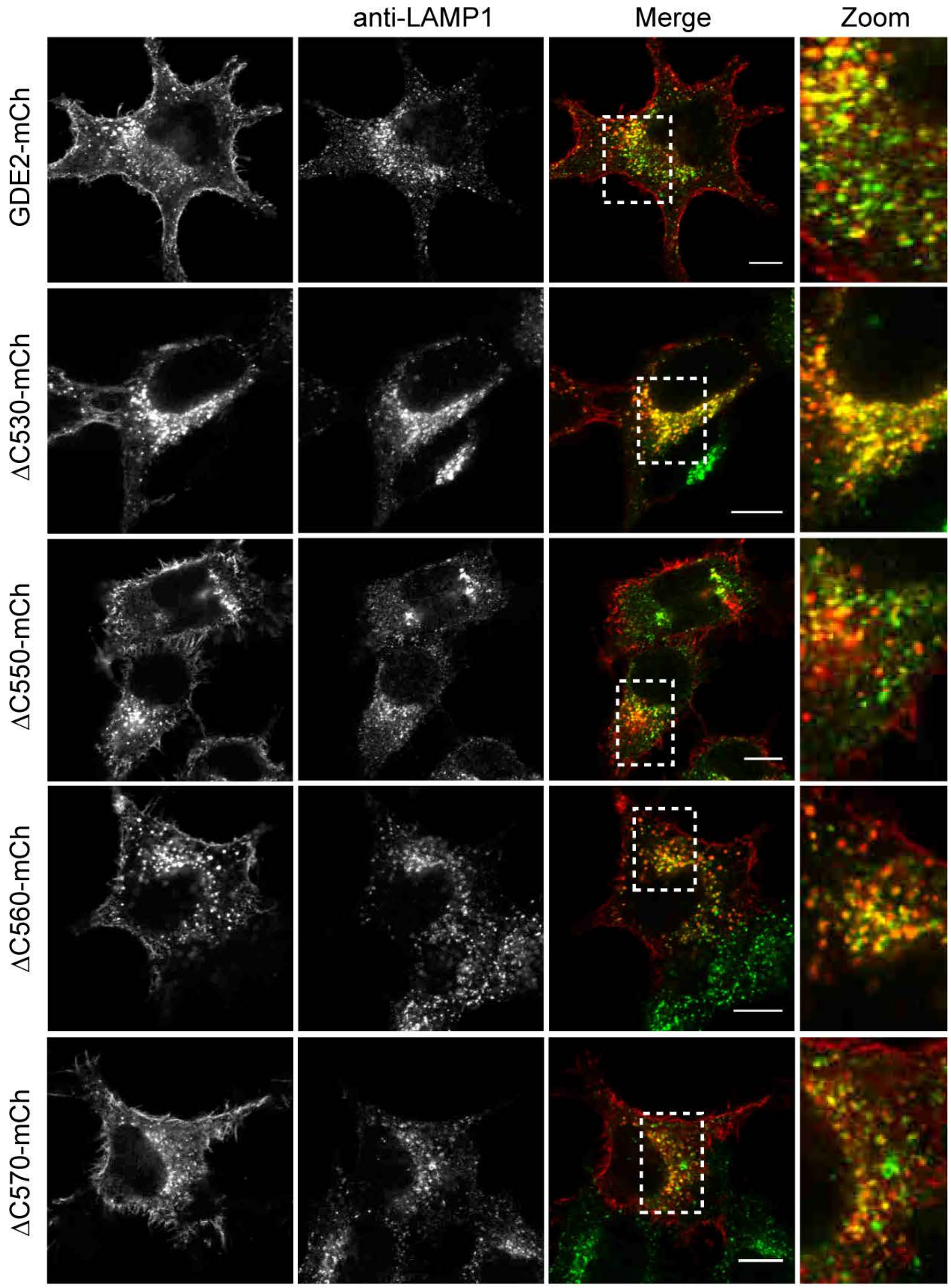
anti-LAMP1
Merge
Zoom
GDE2-mCh
ΔC530-mCh
ΔC550-mCh
ΔC560-mCh
ΔC570-mCh

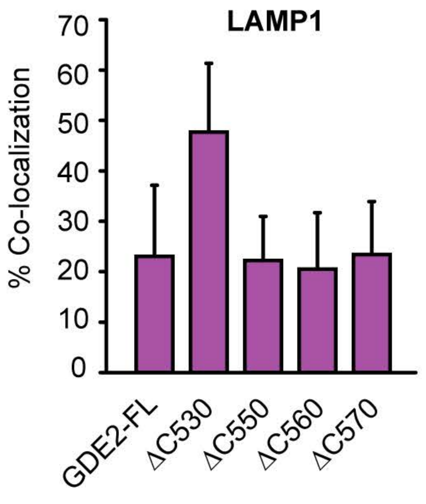
LAMP1
% Co-localization
0
10
20
30
40
50
60
70
GDE2-FL
ΔC530
ΔC550
ΔC560
ΔC570

Figure S3. Lysosomal localization of GDE2 and its truncation mutants.
N1E-115 cells expressing the indicated GDE2-mCh constructs were immunostained for LAMP1, using LAMP1-specific antibody. Bar, 10 μm. Lower panel, quantification of GDE2-LAMP1 co-localization. Data represent the median ± SEM (error bars) of co-localization.

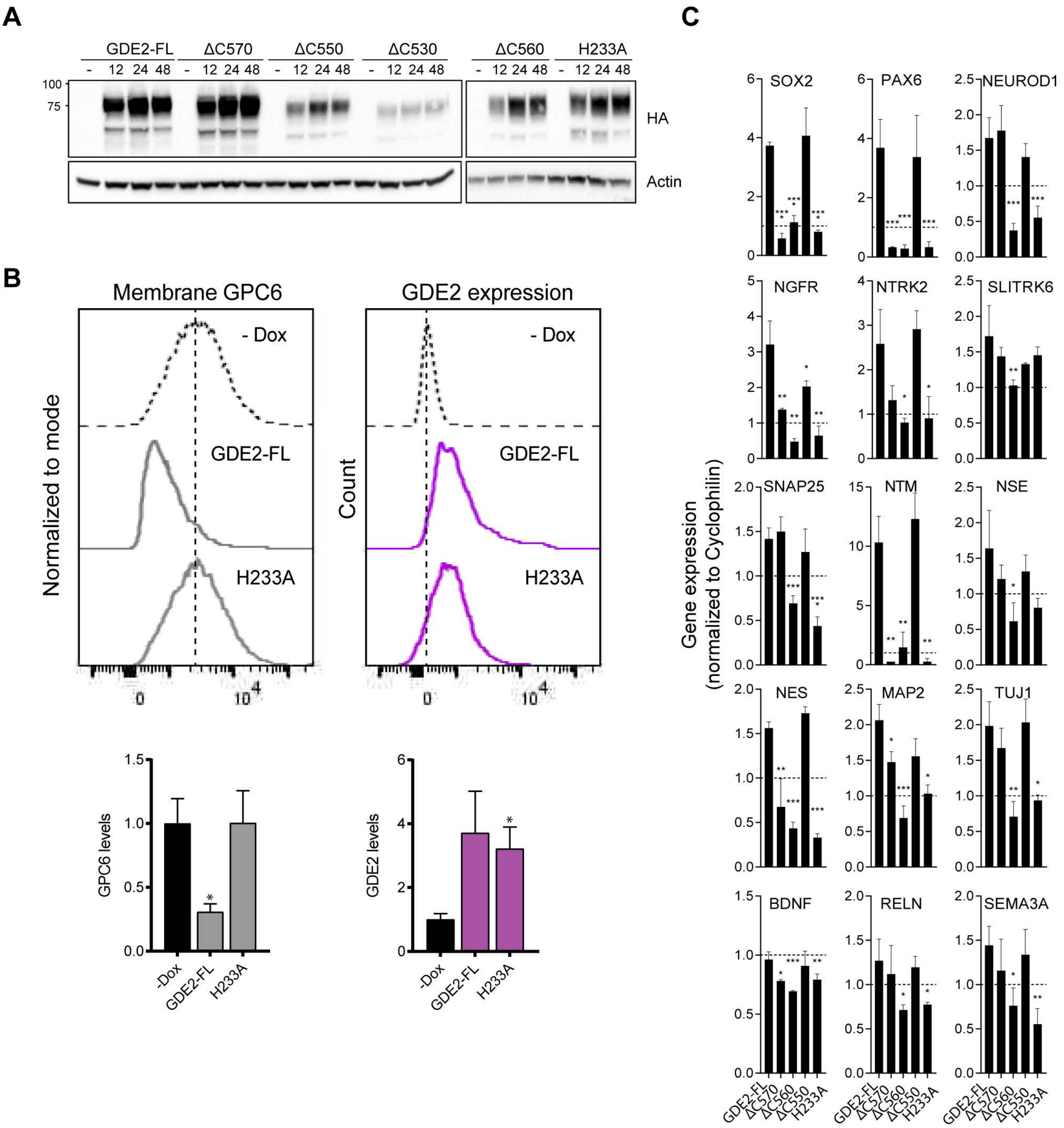

Figure S4. Expression and localization of GDE2 and truncation mutants in Dox-treated SH-SY5Ycells.

A. Western blots showing expression of the indicated GDE2-HA constructs after 24 and 48 hrs of Dox treatment. Actin was used as loading control. See also **Fig. 3A**.

B. Control experiment showing that GDE2(H233A) at the cell surface is inactive towards GPC6. Cell surface expressions were determined by FACS. Data show the median ± SEM (error bars) from triplicate measurements taken from two independent experiments. *p<0.05, paired t test. See also **Fig. 3C.**

C. Induction of neuronal differentiation genes upon Doxycycline induction (48 hrs) of the indicated GDE2 constructs, as determined by qPCR. Data represent the average value of triplicate biological measures ± SEM (error bars). *p<0.05, **p<0.01, ***p<0.001, ****p<0.0001 by unpaired t test. See also **Fig. 3D**.

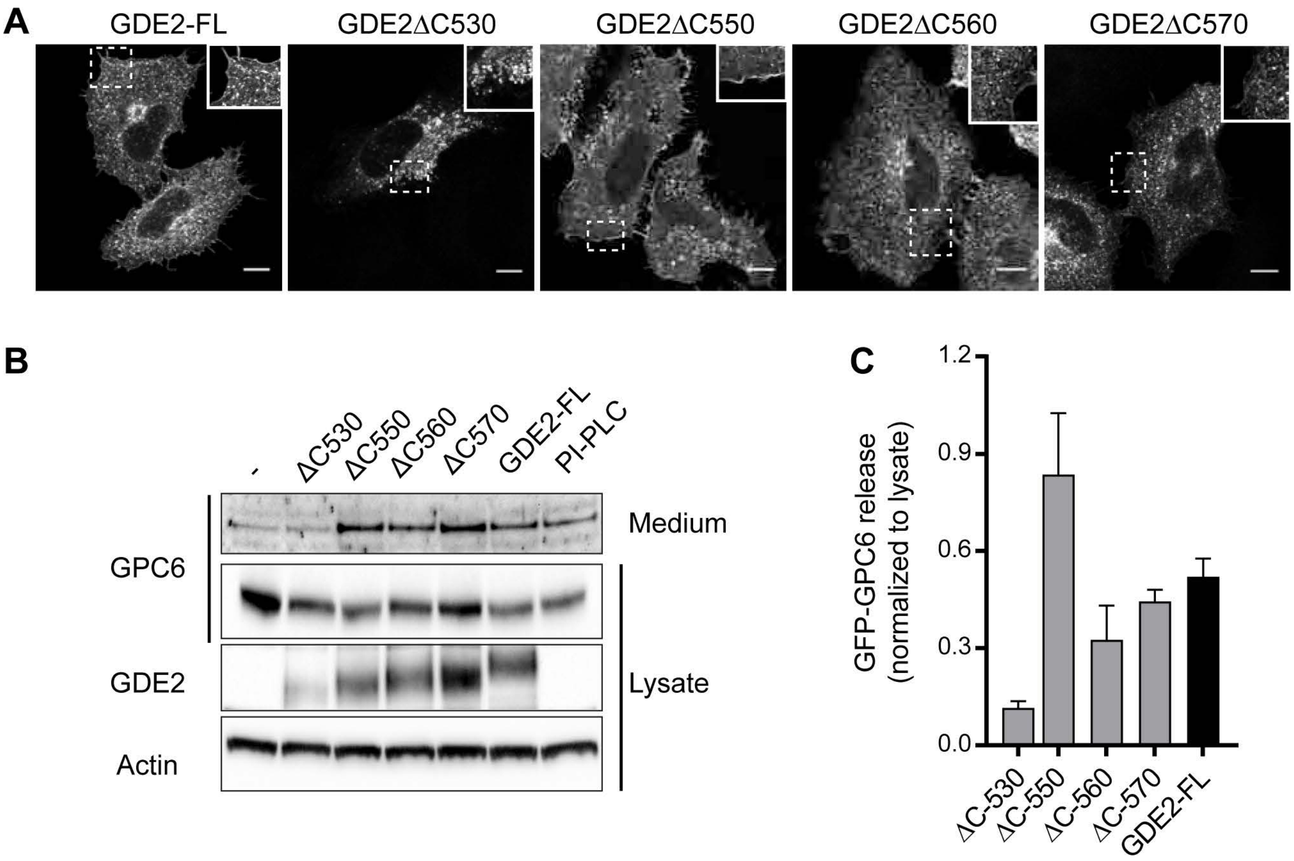

Figure S5. GDE2-induced GPC6 shedding in HeLa cells.

A. Localization of GDE2-HA and its truncation mutants in HeLa cells. Subcellular localizations were similar to those in neuronal cells. Bar, 10 µm.

B. HeLa cells were co-transfected with GFP-GPC6 and GDE2-HA. After 24 hrs, cells were serum-starved and GPC6 was allowed to accumulate in the medium. Bacterial PI-PLC was used as control.

C. Quantitated bands of released GPC6 normalized to its total expression in the cell lysate.

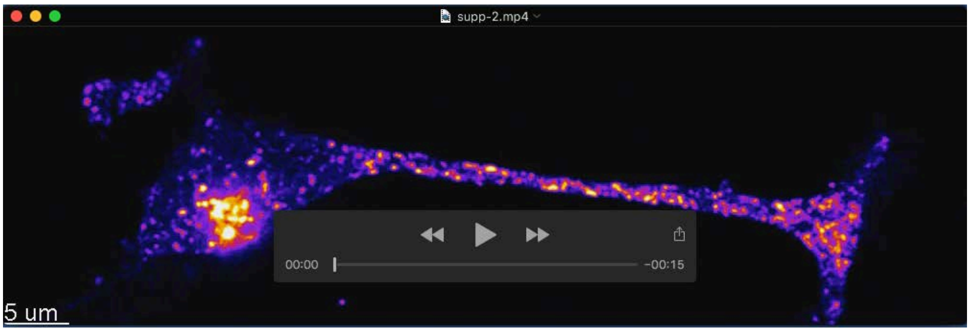

Movie 1. **Live-imaging of GDE2 intracellular trafficking in SH-SY5Y cells.**
A single cell expressing eGFP-tagged GDE2 was imaged on a Leica SP8 confocal microscope using a 63x oil immersion objective at ~8 frames per min. Note the rapid motility of vesicular GDE2 and its bi-directional movement towards and from the neurite ending. Scale bar and timing as indicated. Image size, 81 x 33 μm.

Table S1. Primers used for qPCR

Gene	Forward primer 5'-3'	Reverse primer 5'-3'
BDNF	TGGCTGACACTTTCGAACAC	GGACCCTCATGGACATGTTT
Cyclophilin	CATCTGCACTGCCAAGACTGA	TTGCCAAACACCACATGCTT
MAP2	CCAATGGATTCCCATACAGG	TCCTTGCAGACACCTCCTCT
NES	AGGTGGCCACGTACAGGAC	TCTCAAGGGTAGCAGGCAAG
NEUROD1	CCGACAGAGCCCAGATGTAGTTCTT	GCCCCAGGGTTATGAGACTATCACT
NGFR	GGGCCTCGTGTTCTCCTG	CAGGGATCTCCTCGCACTC
NSE	CTGATGCTGGAGTTGGATGG	CCATTGATCACGTTGAAGGC
NTM	GTCTCTCAGGCTGCTGTTCC	ATCCAGGCACCACTTGTCAT
NTRK2	CGAATCTCCAACCTCAGACC	CCCCATTGTTCATGTGAGTG
PAX6	CAGCTCGGTGGTGTCTTTGT	ACTTGAACTGGAACTGACAC
RELN	CCACAGGCCTTAATACAACAACAG	GGGTCTGAATAACTAAAGCGACATGA
SEMA3A	TTGTCTGTCTTTTCTGGGGAGT	TGTGATCCTTTGCTCCAACA
SLITRK6	AATAACCCATCCATGCCAAC	AACCACTATCCCTGCAGCAC
SNAP25	AGTTGGCTGATGAGTCGCTG	TGAAAAGGCCACAGCATTTC
SOX2	ATGGGTTCGGTGGTCAAGT	GGTAGTGCTGGGACATGTGA
TUJ1	CCTGGAACCCGGAACCAT	AGGCCTGAAGAGATGTCCAAAG

Journal of Cell Science • Supplementary information
